# Life Course Impact of School-Based Promotion of Healthy Eating and Active Living to Prevent Childhood Obesity

**DOI:** 10.1371/journal.pone.0102242

**Published:** 2014-07-15

**Authors:** Bach Xuan Tran, Arto Ohinmaa, Stefan Kuhle, Jeffrey A. Johnson, Paul J. Veugelers

**Affiliations:** 1 School of Public Health, University of Alberta, Edmonton, Alberta, Canada; 2 Department of Pediatrics, Obstetrics & Gynecology, Dalhousie University, Halifax, NS, Canada; Scientific Directorate, Bambino Hospital, Italy

## Abstract

**Background:**

The Alberta Project Promoting active Living and healthy Eating in Schools (APPLE Schools) is a comprehensive school health program that is proven feasible and effective in preventing obesity among school aged children. To support decision making on expanding this program, evidence on its long-term health and economic impacts is particularly critical. In the present study we estimate the life course impact of the APPLE Schools programs in terms of future body weights and avoided health care costs.

**Method:**

We modeled growth rates of body mass index (BMI) using longitudinal data from the National Population Health Survey collected between 1996–2008. These growth rate characteristics were used to project BMI trajectories for students that attended APPLE Schools and for students who attended control schools (141 randomly selected schools) in the Canadian province of Alberta.

**Results:**

Throughout the life course, the prevalence of overweight (including obesity) was 1.2% to 2.8% (1.7 on average) less among students attending APPLE Schools relative to their peers attending control schools. The life course prevalence of obesity was 0.4% to 1.4% (0.8% on average) less among APPLE Schools students. If the APPLE Schools program were to be scaled up, the potential cost savings would be $33 to 82 million per year for the province of Alberta, or $150 to 330 million per year for Canada.

**Conclusions:**

These projected health and economic benefits seem to support broader implementation of school-based health promotion programs.

## Introduction

Obesity affects the health of Canadians and costs the nation approximately $1.27 to 11.08 billion per year in health care [1]. A myriad of psychological and physical consequences hamper obese individuals to function as healthy and productive members of the society. The physical consequences include chronic and fatal diseases such as cardiovascular disease, type 2 diabetes, and various cancers [2], [3].

Poor eating habits and sedentary lifestyles are the established risk factors for obesity. Promotion of healthy eating and active living is considered to be most effective when targeting childhood years [4], [5]. In the Canadian province of Alberta, we recently demonstrated the feasibility and effectiveness of a school-based program in preventing childhood obesity [6]. This Alberta Project Promoting active Living and healthy Eating in Schools (APPLE Schools) is a comprehensive school health program that started as a pilot in 2008 in 10 elementary schools. The intervention involved a full-time School Health Facilitator in each school for implementing healthy eating and active living policies, practices and strategies while engaging stakeholders, including parents, staff and the community. School Health Facilitators contributed to the schools' health curriculum, and organized nutrition programs such as cooking clubs and healthy breakfast, lunch and snack programs, after school physical activity programs, walk-to-school days, community gardens, weekend events and circulated newsletters. By 2010 the eating habits and physical activity levels of students attending APPLE Schools had significantly improved whereas the prevalence of obesity had declined relative to their peers attending other Albertan schools [6]. These findings are consistent with other school-based programs internationally that took a comprehensive approach to promoting healthy eating and active living [5], [7]–[9].

It is recognized that health status in early periods of life form the foundation for a healthier life course and that obesity in childhood often persists into adulthood. However, to date little is known about the long-term implications of successful prevention of childhood obesity [10]. Public health decision makers wish to be informed on the long-term health benefits and financial implications of these prevention programs. Guyer et al acknowledged the need for well-designed longitudinal studies to determine the importance of childhood interventions for health outcomes in adulthood, or in other words, to estimate the impact of early interventions on the life-course of the youngsters who had been subjected to these intervention (a life-course approach) [10]. The purpose of this study is to estimate the life course impact of the APPLE Schools program in terms of future body weight status and avoided health care costs.

## Materials and Methods

This study has been reviewed and approved by the Health Research Ethics Board of the University of Alberta, Edmonton, Alberta, Canada.

### Data source and statistical analysis

We previously published that the prevalence of obesity among grade five students (typically 10 or 11 years of age) attending APPLE Schools reduced 2.2% between 2008 and 2010 as compared to a 2.8% increase in the prevalence of obesity among grade fivers attending other schools [6]. This difference is equivalent to reductions of 0.26 and 0.17 kg/m^2^ in body mass index (BMI) per year among girls and boys, respectively, participating in APPLE Schools programs. These intervention benefits were used as the starting points of life course BMI trajectories to estimate the life course impact of the APPLE Schools intervention.

For the purpose of determining longitudinal BMI trajectories throughout the life course, we accessed longitudinal data of persons of all ages who participated in the Canadian National Population Health Surveys (NPHS). The NPHS included longitudinal assessment of 17,276 persons selected from each of the 10 Canadian provinces [11]–[13]. The data were anonymized and we accessed and analyzed the data at the Research Data Center of the University of Alberta, Edmonton, AB, Canada. The NPHS employed a stratified two-stage sample design based on the Labour Force Survey in all provinces except for the province of Québec, where the another survey, the Enquête Sociale et de Santé, was used. Participants were followed up and interviewed every two years (cycles) using a common set of health questions. Follow up response rates ranged from 92.8% in cycle 2 to 70.7% in cycle 8. Data collection continued when participants were institutionalized in a long-term care facility and included verification of vital status. Data collection at baseline (cycle 1 in 1994/1995) was through in-person interviews, and in subsequent cycles through telephone interviews [11]. To minimize systematic differences in data collection, we excluded cycle 1 from the present analysis. Cycle 2 to 8 provide longitudinal data for 1996 to 2008 that included self-reported heights and weights. We restricted our analyses to observations of individuals in the age range of 11 to 70 years, as 11 is the typical age students reach while in grade five (i.e., the grade level of the assessment of the APPLE Schools program effectiveness). Age 70 was set as the upper labour age in light of assessing potential economic implications. We used standards set by the World Health Organization to classify adult body weight categories; underweight (BMI <18.5), normal weight (BMI ≥18.5 to <25), overweight (BMI ≥25 to <30), and obesity (BMI ≥30).

We applied growth curve models to the longitudinal NPHS data to quantify individual changes in BMI over time. These BMI trajectories were estimated for 5 different age period: 11 to <23, 23 to <35, 35 to <47, 47 to <59, and 59 to 70 years of age. This staggered modeling approach provided flexibility to the full life course BMI trajectory and improved our ability to estimate the models since each NPHS participant was tracked for 14 years over 7 NPHS cycles. The growth curve models describe BMI changes in one age category as a function of the development of BMI in the previous age category. In other words, for each age category, the growth curve model estimates the changes in BMI based on the BMI starting value in that particular age category. For example, we estimated the BMI growth for the age 11 to less than 23 years using the BMI at age 11 years. This would provide a projected BMI at age 22.99 years, which would then be the starting value for the estimation of the BMI growth in the subsequent age category, 23 to less than 34 years, and so on.

The model selection was purposive, and we applied an analytical procedure that had been used in previous studies and shown to be robust in estimating trajectories of BMI of individuals using this data set [14], [15]. The models were adjusted for survey sampling weights, sex, body weight status, and calendar year. We further considered interaction terms of sex and age, and of sex and the quadratic form of age [14]. Body weight status (at the beginning of each age category) was considered as a random effect in these models, as were sex as well as intercept and linear slope [14]. The growth curve models were considered to have an unstructured covariance matrix [14]. We computed Bosker/Snijders and Bryk/Raudenbush R-square values for mixed models with two levels.

To project the life course BMI trajectories of grade five students in Alberta, we applied the parameters from the above described growth curve models to overweight and obesity prevalence rates of Alberta [6]. These prevalence rates originated from a population-based survey including 3,398 grade five students from 141 randomly selected schools from across Alberta in 2010 [6]. We then repeated the projection of the life course BMI trajectories adjusting the starting prevalence rates for the reduction in BMI resulting from the APPLE Schools program. The differences between the two models then represent the potential life course impact of the APPLE School program on the projected BMI status.

Projection of health care cost savings by reduction in the prevalence of overweight and obesity was estimated by multiplying the total direct health care cost for obesity by the proportion of overweight and obese cases prevented by the intervention. An updated estimation by Anis et al showed that the annual direct health care cost of overweight and obesity in Canada was $ 6 billion in 2006 [16]. We assumed that this cost remained unchanged overtime and for every overweight and obese case that we prevented, we avoided the costs for health conditions related to obesity.

## Results

**Figure 1:**
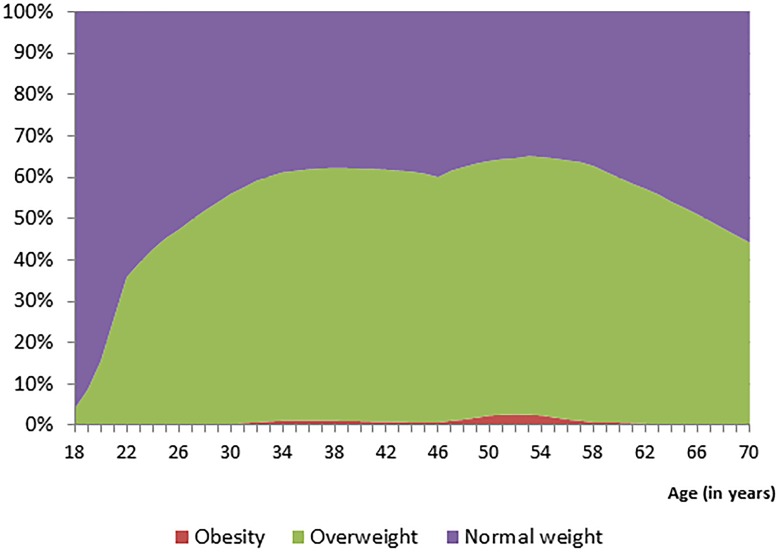
Life course weight status projections of normal weight grade five students.

**Figure 2:**
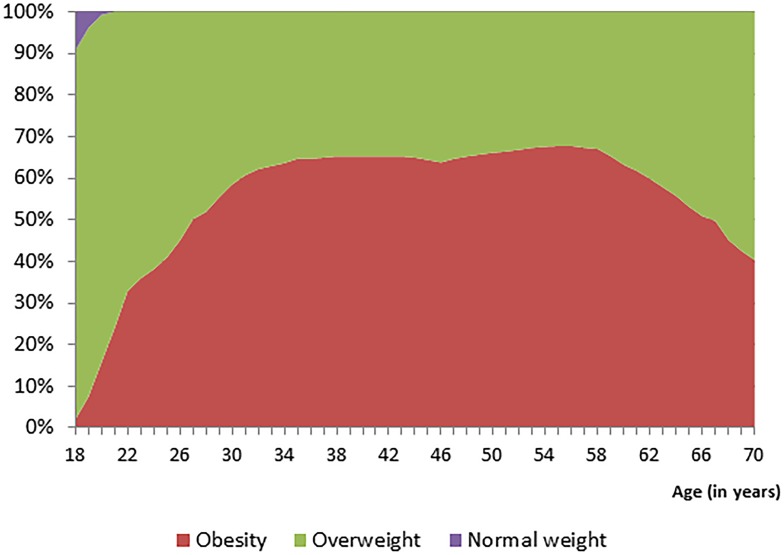
Life course weight status projections of overweight grade five students.

**Figure 3:**
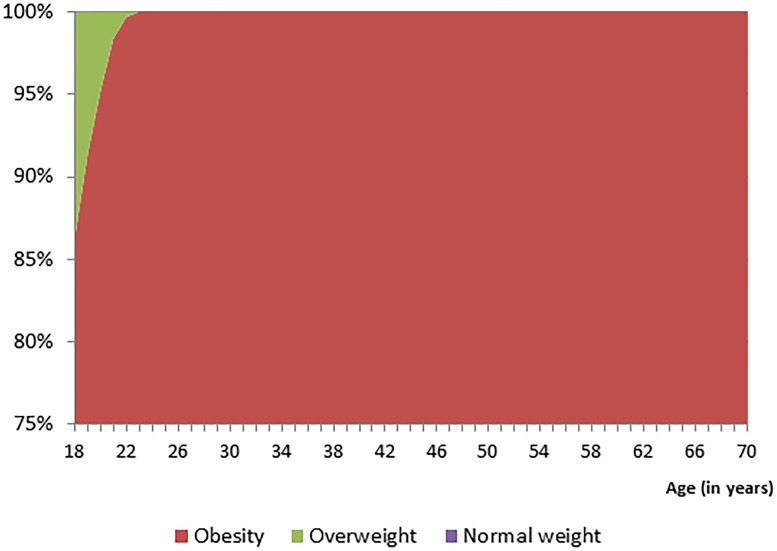
Life course weight status projections of obese grade five students.

Table 1 presents the parameter estimates for each of the 5 age-specific growth curve models used to project life course changes in BMI for grade five students in Alberta. Figure 1 presents the projected body weight status for grade five students with normal weight using the parameter estimates of Table 1. Approximately 40% of normal weight youth are estimated to progress to overweight by the time they turn 25 years of age. This percentage will further increase to 60% by the time they turn 35 years of age. Figure 2 depicts the projected body weight status for grade five students who were overweight and shows that 60% will progress to obesity by the time they turn 30 years of age. Very few overweight youth enter adulthood as normal weight (Figure 2). Lastly, figure 3 shows that nearly all obese youth progresses to obese adults.

**Table 1:** Growth curve modeling of BMI for five age categories of participants of the Canadian National Population Health Survey.

	Age: 11,<23	Age: 23,<35	Age: 35,<−47	Age: 47,<59	Age: 59,<71
Boy	**7.767**	**15.442**	**29.673**	**2.766**	−0.300
Girl	**4.800**	**13.915**	**11.684**	9.631	−1.674
Boy×Age	**1.285**	**0.571**	−0.201	**0.903**	0.903
Girl×Age	**1.701**	0.569	**0.604**	0.562	0.903
Boy×Age∧2	**−0.023**	−0.008	0.003	**−0.008**	−0.008
Girl×Age∧2	**−0.040**	−0.008	−0.007	−0.005	−0.007

Year					
1996	reference	reference	reference	reference	reference
1998	−0.134	**0.207**	**0.220**	0.117	0.029
2000	−0.013	**0.358**	**0.589**	**0.380**	**0.328**
2002	0.162	**0.811**	**0.882**	**0.683**	**0.647**
2004	0.367	**0.911**	**0.978**	**0.643**	**0.673**
2006	0.058	**1.002**	**1.232**	**0.868**	**0.923**
2008	0.029	**1.139**	**1.299**	**1.011**	**1.082**
R2 coefficient	0.33	0.12	0.08	0.04	0.02

Note: Figures in bold represent statistically significant estimates (p < 0.05).

**Figure 4:**
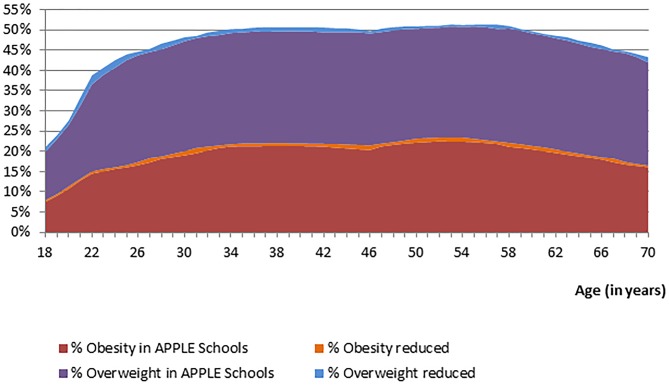
Life course weight status projections for the grade five students attending the APPLE Schools program and those who are not. Figure 4 footnote: Purple represents the percentage of students attending APPLE Schools who are projected to become overweight; Blue represents the percentage students attending other Alberta schools who are projected to become overweight; Red represents the percentage of students attending APPLE Schools who are projected to become obese; Orange represents the percentage students attending other Alberta schools who are projected to become obese.

**Figure 5:**
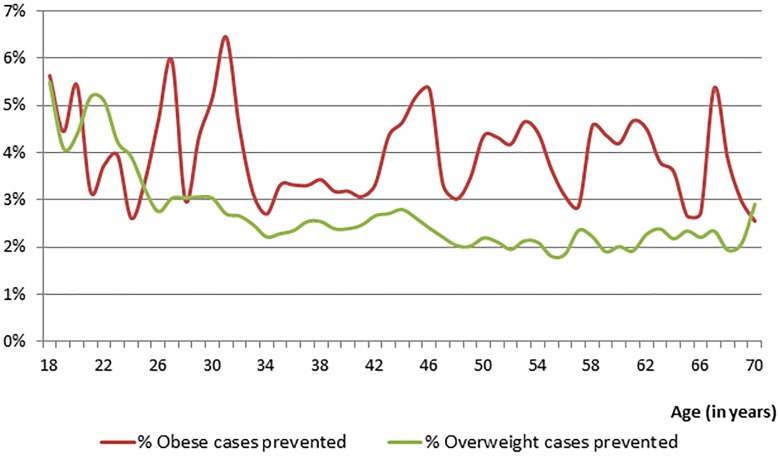
Life course projections of the percentage prevented overweight and obese cases.

When applying the grow curves to grade five students attending APPLE Schools, we estimated that for every unit increase in BMI at age 11, 23, 35, 47, and 59, the BMI growth rate over the five corresponding age-specific periods increased by 0.82, 0.890, 0.969, 0.930, and 0.863 kg/m^2^ respectively (p<0.05). Comparing the estimates of the growth curves applied to students attending APPLE Schools and attending other Alberta schools, we quantified the benefits of the APPLE Schools program throughout the students' lifetime. This is presented in Figure 4. The lifetime prevalence of overweight (including obesity) was 1.2% to 2.8% (1.7 on average) less among students attending APPLE Schools relative to those attending other Alberta schools. The prevalence of obesity was 0.4% to 1.4% (0.8% on average) less among students attending APPLE Schools students relative to those attending other Alberta schools (Figure 4). We estimated that 2% to 5.5% of overweight cases and 3% to 6.5% of obesity cases could be prevented through the APPLE Schools program (Figure 5).

**Figure 6:**
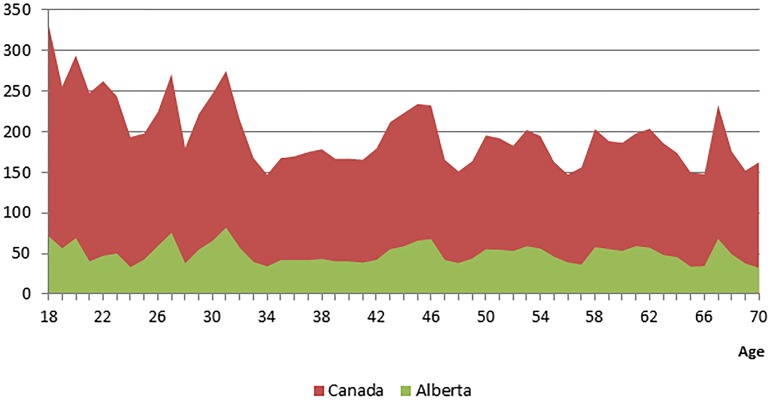
Life course projections of avoided health care costs for Canada and the province of Alberta (in million dollars).

If this program were to be scaled up to Canada that spends approximately 6 billion dollars for health care for people with excess body weight, the potential cost savings would be 150 to 330 million dollars per year (Figure 6) [16]. Similarly, if this program were to be scaled up to Alberta, it could save 33–82 million dollars for obesity-related health care in Alberta (Figure 6). The avoided health care costs were in average higher at younger ages (Figure 6).

## Discussion

We projected of body weight trajectories of youth in Alberta and forecasted that more than two thirds is likely to develop excess body weight at some point in their lives. We further modeled the long-term benefits of the APPLE Schools intervention and forecasted that the prevalence of overweight (including obesity) among students attending APPLE Schools is 2% to 6% less relative to the prevalence among their peers who are attending other schools in Alberta. With a nationwide implementation of the APPLE Schools program, this could result in 150 to 330 million dollars per year in cost savings due to avoided health care services.

We forecasted that more than two thirds of current youth is likely to become overweight or obese at some point in their lives. This seems higher that the forecasts by Kuhle [17] who reported that 45% of youth would have excess body weight by 2006 and 55% by in 2026. Forecasts in the US had revealed that in 2010 the obesity prevalence of sex and racial subgroups ranges from 33 to 55% and that the national prevalence of obesity is expected to increase to 51% by 2030 [18], [19].

The present study is the first to follow a life course approach for the purpose of quantifying the impact on adulthood obesity of school-based promotion of healthy eating and active living. Our findings are consistent with the existing evidence that interventions at an early age are effective in influencing body weight status later in life and that school-based prevention programs may therefore be cost effective [5], [20]–[26]. In the United States, Wang et al. developed a progression model to project the long-term benefits of a school based intervention and reported a 1% reduction in overweight and obese and $586 million in cost savings [27]. We estimated direct health care costs savings of $150 to 330 million per year in Canada, based on the prevention of 2% to 6% of the projected cases of overweight and obesity. If these estimates hold true, it would seem that small reductions in childhood obesity prevalence translate into large costs savings at the population level, and thus school programs and other initiatives that can further reduce overweight prevalence rates will further contribute to program effectiveness and cost savings.

Body weight in childhood is an established predictor of body weight at a later age [20], [28]–[35]. For example, Magarey et al had tracked the weight status of Finnish children and identified weight status at age 6 as a strong predictor of weight status in adulthood [28], and Starc and Strel tracked 4,833 Slovenian children and found that those who were obese at age 18 years, 40% of males and 48.6% of females had been obese at 7 years [31]. It is for this reason that we had considered early body weight in our growth curves, and our analyses confirmed the importance of body weight for growth in body weight. However, reduction in the prevalence of excess body weight was not the only benefit reported for APPLE Schools. We had also reported benefits to healthy eating and active living [6], that were achieved throughout a comprehensive approach that improved students' knowledge levels, attitudes, self-efficacy and leadership skills related to making healthy choices. Where knowledge, attitudes and life skills may persist over the life course of APPLE Schools graduates, they may contribute to healthier choices later in life and herewith to more prevention of excess body weight. The health and costs benefits revealed in this study may therefore not have fully captured the potential impacts of the APPLE Schools program, and comprehensive school health programs in general.

A strength of the present study is that it was based on large established national and provincial studies as well as a feasible school-based intervention that will improve the generalizability of the findings. More over, we followed a life course approach to provide insight into the future health benefits and cost implications of interventions. Where students' heights and weights were measured in APPLE Schools and control schools, heights and weights in the longitudinal NPHS were obtained through self-report. Self-report of height and weight is prone to error which we acknowledge as a study limitation. Other study limitations may relate to the use of administrative health care databases for the purpose of estimating avoided health case costs. Furthermore, projections assume that future developments follow patterns similar to the patterns in the observations on which the projection models are based. We acknowledge that future patterns may deviate from observed patterns which, in turn, may affect our estimates.

To conclude, preventing childhood obesity during their school years is forecasted to reduce obesity in adulthood which may lead to substantial savings in future health care costs at population level. Youth with healthy weights are less likely to develop overweight and obesity through their lives. Also, healthy habits and skills acquired in childhood may lend for life long healthy behaviors that further reduce the likelihood of weight gain [36]. Potential cost savings should encourage the allocation of resources towards school-based promotion of healthy eating and active living.
